# Supplementary material for: Analysis of PPARγ Signaling Activity in Psoriasis
Source: Int J Mol Sci. 2021 Aug 10;22(16):8603. doi: 10.3390/ijms22168603 (PMC8395241; doi:10.3390/ijms22168603)
Supplement: Supplementary file 1 [file ijms-22-08603-s001.zip › Supplemental materials_Analysis of PPARg signaling activity in psoriasis/Pathway models/Models images and html files/Differentiation of psoriatic T cells/6378.html]

IL12RB2


# Protein IL12RB2

|  |  |
| --- | --- |
| URN | urn:agi-llid:3595 |
| Total Entities | 0 |
| Connectivity | 478 |
| Name | IL12RB2 |
| Description | interleukin 12 receptor, beta 2 |
| Notes | The protein encoded by this gene is a type I transmembrane protein identified as a subunit of the interleukin 12 receptor complex. The coexpression of this and IL12RB1 proteins was shown to lead to the formation of high-affinity IL12 binding sites and reconstitution of IL12 dependent signaling. The expression of this gene is up-regulated by interferon gamma in Th1 cells, and plays a role in Th1 cell differentiation. The up-regulation of this gene is found to be associated with a number of infectious diseases, such as Crohn's disease and leprosy, which is thought to contribute to the inflammatory response and host defense. Several transcript variants encoding different isoforms and non-protein coding transcripts have been found for this gene. [provided by RefSeq, Apr 2012] |
| Primary Cell Localization | Plasma membrane |
| Class | Receptor |

---

|  |  |
| --- | --- |
| Pathway | CD8+ T-Cell Activation |
|  | Th1-Cell Differentiation |
|  | Th17-Cell Differentiation |
|  | T-lymphoid precursor cell -> CD8+ Naive T-cell Surface Expression Markers |
|  | Th1-Cells Activation and Proliferation in Atherosclerosis |
|  | Lymphocyte Mediated Myocardial Injury in Myocarditis |
|  | Atopic Dermatitis |
|  | T-Cells Differentiation Block in Psoriasis |
|  | Th17-Cell Function in Systemic Lupus Erythematosus |
|  | Th1-Cell Function in Systemic Lupus Erythematosus |
|  | T-Cell Cytotoxic Response against Melanocytes in Vitiligo |
|  | Toll-like Receptors in beta-Cell |
|  | CD4+T-Cell Response in Celiac Disease |
|  | Th1-Cell Activation in Crohn's Disease |
|  | Th17-Cell Activation in Crohn's Disease |
|  | Follicular Lymphoma |
|  | Il17 Signaling Related Neutrophilia in Asthma |
|  | Th17-Cell Differentiation in Asthma |
|  | Th17-Cell and Th1 Immune Responsein Psoriatic Arthritis |
|  | Th17-Cell Numbers Reduction in HIV |
|  | IL12R -> STAT Signaling |
|  | IL23R -> STAT3/NF-kB Signaling |
|  | IL12R -> NF-kB/NFATC Signaling |
|  | IL12B Expression Targets |
|  | CD40 -> Expression Targets in Thymus |
|  | PPAR Psoriasis |
|  | 1\_Differentiation of psoriatic T cells |
|  | 1\_Antigen-presenting cells promote islet dysfunction and immune system activation in T1D |
|  | Differentiation of psoriatic T cells |

---

|  |  |
| --- | --- |
| Group | Genes Frequently Hypermethylated in Follicular Lymphoma |

---

|  |  |
| --- | --- |
| MedScan ID | 3595 |

---

|  |  |
| --- | --- |
| LocusLink ID | 3595 |
|  | 16162 |
|  | 171334 |

---

|  |  |
| --- | --- |
| Alias | interleukin IL-12 receptor beta 2 |
|  | interleukin 12 receptor, beta II |
|  | interleukin-12 receptor subunit beta-2 |
|  | IL12rb2 |
|  | IL-12 receptor beta II |
|  | interleukin IL12rb2 |
|  | OTTHUMP00000010662 |
|  | Ifnm |
|  | interleukin 12 receptor beta 2 subunit |
|  | interleukin IL-12 receptor beta II |
|  | IL-12 receptor beta 2 |
|  | IL-12R subunit beta-2 |
|  | interferon response to microorganisms |
|  | RP11-102M16.1 |
|  | A930027I18Rik |
|  | A930027I18 |
|  | IL-12 receptor subunit beta-2 |
|  | RIKEN cDNA A930027I18 gene |
|  | I12S\_HUMAN |
|  | interleukin 12 receptor, beta 2 |
|  | interleukin IL-12R-beta2 |
|  | IL-12R-beta2 |
|  | interleukin-12 receptor beta-2 chain |
|  | IL-12RB2 |
|  | IL-12R-beta-2 |

---

|  |  |
| --- | --- |
| GO ID | 0019955 |
|  | 0004896 |
|  | 0019901 |
|  | 0007166 |
|  | 0032609 |
|  | 0035722 |
|  | 0070757 |
|  | 0018108 |
|  | 0008284 |
|  | 0032729 |
|  | 0032496 |
|  | 0009897 |
|  | 0005887 |
|  | 0005886 |
|  | 0043235 |
|  | 0034097 |
|  | 0016021 |
|  | 0016020 |
|  | 0019221 |
|  | 0045622 |
|  | 0004872 |
|  | 0007165 |

---

|  |  |
| --- | --- |
| KEGG ID | hsa:3595 |
|  | mmu:16162 |
|  | rno:171334 |

---

|  |  |
| --- | --- |
| Organism | Homo sapiens {Organism urn:agi-taxid:9606} |
|  | Mus musculus {Organism urn:agi-taxid:10090} |
|  | Rattus norvegicus {Organism urn:agi-taxid:10116} |
|  | Mus musculus |
|  | Homo sapiens |
|  | Rattus norvegicus |

---

|  |  |
| --- | --- |
| Mouse chromosome position | 6 30.81 cM |
|  | 6 30.1 cM |

---

|  |  |
| --- | --- |
| OMIM ID | 601642 |

---

|  |  |
| --- | --- |
| Rat chromosome position | 4q31 |

---

|  |  |
| --- | --- |
| Hugo ID | 5972 |
|  | HGNC:5972 |

---

|  |  |
| --- | --- |
| Human chromosome position | 1p31.3 |
|  | 1p31.3-p31.2 |

---

|  |  |
| --- | --- |
| Swiss-Prot Accession | B4DGA4 |
|  | Q99665 |
|  | A0A0A0MTN7 |
|  | B7ZB60 |
|  | Q99665.1 |
|  | D3Z6H5 |
|  | P97378 |
|  | P97378.1 |
|  | F1LRH7 |
|  | B1AN98 |
|  | B7ZKL9 |
|  | F5H7L6 |
|  | Q2M3V3 |
|  | B9EHD9 |
|  | Q9Z0Z1 |

---

|  |  |
| --- | --- |
| GenBank ID | NC\_000001 |
|  | NR\_047584 |
|  | NR\_047583 |
|  | XM\_005270825 |
|  | XP\_005270882 |
|  | XM\_011541384 |
|  | XP\_011539686 |
|  | XM\_006710617 |
|  | XP\_006710680 |
|  | XM\_005270827 |
|  | XP\_005270884 |
|  | XM\_017001203 |
|  | XP\_016856692 |
|  | XM\_005270828 |
|  | XP\_005270885 |
|  | XM\_017001204 |
|  | XP\_016856693 |
|  | NM\_001258214 |
|  | NP\_001245143 |
|  | NM\_001319233 |
|  | NP\_001306162 |
|  | NM\_001559 |
|  | NP\_001550 |
|  | NM\_001258216 |
|  | NP\_001245145 |
|  | NM\_001258215 |
|  | NP\_001245144 |
|  | XM\_011541383 |
|  | XP\_011539685 |
|  | NG\_032977 |
|  | AF349574 |
|  | AL358512 |
|  | AL389925 |
|  | AY640177 |
|  | AAT45456 |
|  | CH471059 |
|  | EAX06499 |
|  | EAX06500 |
|  | AK294494 |
|  | BAG57715 |
|  | AK300020 |
|  | BAG61833 |
|  | AK310837 |
|  | AK316525 |
|  | BAH14896 |
|  | BC104772 |
|  | AAI04773 |
|  | BC104774 |
|  | AAI04775 |
|  | BC143248 |
|  | BC143249 |
|  | AAI43250 |
|  | BC143250 |
|  | BC143252 |
|  | DA223630 |
|  | DB126833 |
|  | U64198 |
|  | AAB36675 |
|  | Q99665 |
|  | NC\_000072 |
|  | XM\_006505617 |
|  | XP\_006505680 |
|  | NM\_008354 |
|  | NP\_032380 |
|  | NM\_001311141 |
|  | NP\_001298070 |
|  | AC130822 |
|  | CH466523 |
|  | EDK98805 |
|  | AK040072 |
|  | BC137745 |
|  | AAI37746 |
|  | U64199 |
|  | AAB36676 |
|  | P97378 |
|  | NC\_005103 |
|  | XM\_006236614 |
|  | XP\_006236676 |
|  | XM\_006236613 |
|  | XP\_006236675 |
|  | NM\_001191750 |
|  | NP\_001178679 |
|  | XM\_008762975 |
|  | XP\_008761197 |
|  | XM\_008762974 |
|  | XP\_008761196 |
|  | XM\_017592423 |
|  | XP\_017447912 |
|  | AC\_000072 |
|  | AABR07060865 |
|  | AAHX01030712 |
|  | AAHX01030713 |
|  | AAHX01030714 |
|  | CH474042 |
|  | EDL91574 |
|  | DQ399740 |
|  | DQ399741 |
|  | DQ399742 |
|  | AF083329 |
|  | AAD16040 |
|  | XM\_011541385 |
|  | XP\_011539687 |
|  | NC\_018912 |
|  | AMYH02001201 |
|  | AMYH02001202 |
|  | AMYH02001203 |
|  | AMYH02001204 |
|  | AMYH02001205 |
|  | XM\_011241226 |
|  | XP\_011239528 |
|  | AC\_000028 |
|  | AAHY01053978 |
|  | AAHY01053979 |
|  | AAHY01053980 |
|  | AAHY01053981 |
|  | AAHY01053982 |
|  | AAHY01053983 |
|  | AAHY01053984 |
|  | AAHY01053985 |
|  | AC\_000133 |
|  | ABBA01074518 |
|  | ABBA01074519 |
|  | CAH69960 |
|  | CAH70407 |
|  | CAH70409 |
|  | NT\_032977 |
|  | NW\_921351 |
|  | NW\_001838579 |
|  | AC\_000044 |
|  | NT\_039353 |
|  | NW\_001030811 |
|  | XM\_231873 |
|  | XP\_231873 |
|  | NW\_047693 |
|  | NW\_001084829 |
|  | XM\_001067506 |
|  | XP\_001067506 |
|  | Q9Z0Z1 |

---

|  |  |
| --- | --- |
| Swiss-Prot ID | I12R2\_HUMAN |
|  | I12R2\_MOUSE |
|  | B1AN98\_HUMAN |
|  | B9EHD9\_MOUSE |

---

|  |  |
| --- | --- |
| Cell Localization | Membrane |
|  | Plasma membrane |

---

|  |  |
| --- | --- |
| Ensembl ID | ENSG00000081985 |
|  | ENSP00000360039.1 |
|  | ENST00000371000.5 |
|  | ENSP00000442443.1 |
|  | ENST00000544434.5 |
|  | ENSP00000445276.2 |
|  | ENST00000541374.5 |
|  | ENSP00000262345.1 |
|  | ENST00000262345.5 |
|  | ENSP00000497959.1 |
|  | ENST00000648487.1 |
|  | ENSMUSG00000018341 |
|  | ENSMUSP00000113267.1 |
|  | ENSMUST00000117441.7 |
|  | ENSMUSP00000010605.3 |
|  | ENSMUST00000018485.3 |
|  | ENSRNOG00000007270 |
|  | ENSRNOP00000009883.5 |
|  | ENSRNOT00000009883.6 |
|  | ENSP00000360039 |
|  | ENST00000371000 |
|  | ENSP00000442443 |
|  | ENST00000544434 |
|  | ENSP00000445276 |
|  | ENST00000541374 |
|  | ENSP00000262345 |
|  | ENST00000262345 |
|  | ENSMUSP00000010605 |
|  | ENSMUST00000018485 |
|  | ENSMUSP00000113267 |
|  | ENSMUST00000117441 |
|  | ENSRNOP00000009883 |
|  | ENSRNOT00000009883 |

---

|  |  |
| --- | --- |
| MGI ID | MGI:1270861 |
|  | 1270861 |

---

|  |  |
| --- | --- |
| RGD ID | 620026 |

---

|  |  |
| --- | --- |
| Unigene ID | Hs.479347 |
|  | Rn.207395 |
|  | Mm.188337 |

---

|  |  |
| --- | --- |
| Homologene ID | 1197 |

---

|  |  |
| --- | --- |
| Shape | Stick-vertex |

---

|  |  |
| --- | --- |
| IPI ID | IPI00438855 |
|  | IPI00438856 |
|  | IPI00124243 |
|  | IPI00479253 |
|  | IPI00881142 |
|  | IPI00358054 |

---
